# Breast Cancer and COVID-19: Challenges in Surgical Management

**DOI:** 10.3390/cancers14215360

**Published:** 2022-10-31

**Authors:** Zoe Petropoulou, Nikolaos Arkadopoulos, Nikolaos V. Michalopoulos

**Affiliations:** 4th Department of Surgery, Medical School, “Attikon” University Hospital, University of Athens, 12462 Athens, Greece

**Keywords:** breast cancer, COVID-19, cancer care, screening, surgery, psychological distress

## Abstract

**Simple Summary:**

The COVID-19 pandemic imposed serious strain on healthcare services and patient management, affecting almost every medical field. Cancer patients underwent and are still facing major modifications and turbulences with regard to their therapeutic courses, with the medical community awkwardly balancing the disease’s menacing nature and their increased vulnerability to novel infection. As the cancer with the highest incidence and prevalence, breast-cancer patients and caregivers were widely affected by the healthcare crisis in multiple domains and ways, leading to rapid adjustments in response, maintaining one aim: to provide safe and uninterrupted cancer care regardless of the resource and communication shortages. This review summarizes the challenges in breast-cancer management and the subsequent alterations in clinical practice. The reflexes and adaptability of the medical community under this massive pressure provide a glimmer of optimism, but the impact of these forced changes and their contribution to this goal still need to be evaluated.

**Abstract:**

The harsh healthcare reality imposed by the COVID-19 pandemic resulted in wide clinical practice alterations, postponements, and shortages, affecting both patients and caregivers. Breast-cancer management, from diagnosis to treatment and follow up, was a field that did not escape such changes, facing a challenging set of obstacles in order to maintain adequate cancer care services while diminishing viral spread among patients and personnel. In this review article, we discuss the impact of the COVID-19 pandemic on several aspects of breast-cancer management, and the subsequent modifications adopted by clinicians, scientific groups, and governments as a response to the novel conditions. Screening and diagnosis, as well as breast-cancer treatment paths—especially surgical interventions—were the most affected domains, while patients’ psychological burden also emerged as a notable consequence. The aftermath of diagnostic and surgical delays is yet to be assessed, while the treatment alterations and the introduction of new therapeutic schemes might signify the opening of a novel era in breast-cancer management.

## 1. Introduction

The COVID-19 pandemic has imposed significant strain on healthcare services, including cancer-care providers. The struggle mostly concerns the fine balance between the risk of virus transmission among vulnerable oncological patients and valuable healthcare personnel, the preservation of human and material resources, the alleviation of facilities’ overload, and the ensuring of high-quality cancer care.

Restrictive measures and social distancing; the redeployment of human resources; and limitations in personal protective equipment, material, and hospital beds revealed the need for adapting to a new, hostile clinical setting, both for patients and clinicians, with subsequent inevitable modifications, delays, or even postponements in many aspects of clinical practice. More specifically, concerning breast-cancer patients, many national committees responded to these unprecedented conditions with omissions of screening and follow-up programs during the overload phases of the pandemic, as well as alterations in the execution of diagnostic and treatment procedures. Major health organizations and professional bodies issued recommendations to provide some of the much needed guidance regarding demanding breast-cancer-patient management from diagnosis to treatment and follow-up during this overwhelming period.

In addition to healthcare systems’ dysfunctions, patients’ psychology and behavior were not spared from the devastating effects of the pandemic, leading to further delays and cancellations in screening or in diagnostic, therapeutic, or follow-up appointments, further compromising the achievement of delivering adequate cancer care in such a rough setting.

In this review article, we identify, summarize, and eventually assess the impact of the COVID-19 pandemic on several stages of breast-cancer management.

## 2. Main Text

### 2.1. Challenges in Diagnosis

To minimize COVID-19 spread, several social distancing measures and transport limitations were imposed in most countries. Compliance with these measures, as well as the fear of getting infected, led a significant number of women to postpone or cancel medical appointments and breast-cancer screening examinations, even in cases where symptoms were present, with a subsequent reduction in the number of patient referrals and overall breast-cancer diagnoses [1,2,3,4]. In addition to the reluctance of the population eligible for screening, many screening programs worldwide were temporarily halted, and primary care appointments, diagnostic imaging, and breast biopsies were reduced in response to augmented clinical demands and a shortage of human and material resources [1,2,4,5,6,7,8]. This practice was also favored by the recommendations published by experts’ consortia [9,10,11]. As expected, the losses in screening and primary care appointments were translated into a significant decrease in breast-cancer incidence and diagnoses during the intense phases of the pandemic, especially for in situ ductal carcinomas (DCIS), early-stage disease, and women aged over 50 years old (the cutoff age for some screening programs), according to some studies [2,5].

The consequences of these diagnostic delays are not yet extensively known, but estimation models based on well-monitored populations predict a possible increase in breast-cancer-related deaths, proportional to the duration of screening cessation [4,8]. Long-term studies should be conducted in order to shed light on this assumption.

Providing screening programs and non-urgent primary and specialized breast-cancer diagnostic services during periods of such overload may not be feasible, but a number of alternatives and modifications can contribute to the effort to stay close to the standard of care. Notably, the use of telemedicine emerged as a useful referral alternative that spares medical resources and provides the opportunity to avoid unnecessary in-person visits, advantages that made it a preferable option among physicians [7]. Furthermore, instead of complete screening and diagnostic-program cessation, detailed planning and information on safety protocols during diagnostic imaging and procedures, as well as the modification of the number of women per screening session, can ease the execution of these programs, achieving safer procedures and fewer patient cancellations.

### 2.2. Challenges in Treatment

Saving valuable medical and facility resources, and mitigating the spread of SARS-CoV-2 while offering breast-cancer patients high-quality treatment close to the standard care, was again the main struggle regarding breast-cancer treatment and decision making. At the beginning of the pandemic, cancer patients, including breast-cancer patients, were considered high-risk patients for COVID-19-related morbidity and mortality, due to disease and chemotherapy-induced immunosuppression [7]. From this point of view, minimizing the risk of COVID-19 exposure by reducing in-person appointments and sessions, avoiding long hospital stays, and limiting the probability of treatment complications and adverse effects and subsequent readmissions was of paramount importance. Additionally, in the context of the healthcare crisis, immense operating-room schedule redistribution and elective-surgery volume reduction occurred, creating another therapeutic obstacle for a significant portion of breast-cancer patients [6,12]. In this setting, the avoidance of undertreatment and providing safe and timely therapeutic options without compromising the outcomes becomes a quite challenging task. A series of therapeutic modifications emerged as a response to the new conditions, rapidly included in breast cancer decision-making guidelines and recommendations issued especially for clinical practice during the pandemic, which were mostly characterized by a shift from surgical and hospitalization-requiring therapies towards conservative, at-home treatments. Notably, the COVID-19 pandemic era was intensely marked by the broad use of neoadjuvant hormonal therapy for hormonal receptor (HR)-positive tumors, both early-stage and locally advanced breast cancer or even DCIS, as a first-line, surgery-sparing treatment for every age group, serving as a major contributor to surgical-load reduction [2,5,6,8,9,12,13,14,15,16].

Surgical practice and treatment were highly affected during the pandemic and especially during the confinement periods, facing a trend that called for operation omissions, careful preoperative patient selection, and surgical-management modifications [5,12].

Non-urgent surgical procedures were postponed, a decision also supported by several professional group recommendations (ACR, ESMO, ACCN, ASBrS, NAPBC, and CoC) [5,6,7,8,10,11,12,14,17,18,19]. Studies stating that surgery delays of up to 12 weeks have no impact on patients’ long-term survival and the rise in neoadjuvant therapies allowed this direction to be implemented in several countries, with 13.6% of surveyed US breast surgeons declaring to have had all of their operations stopped and 100% reporting a reduction in the number of elective surgeries [3,6,12]. In a survey conducted by Rocco et al., including breast surgeons from various countries worldwide, only 4% of the participants reported retaining unchanged operating schedules. From the affected portion, 62% declared reducing their sessions and 34% performing only emergency breast operations [12]. To achieve this reduction, a primary systemic treatment was offered as an alternative to surgery in 48% of the cases diagnosed during the pandemic [12].

To aid patient selection, multidisciplinary team cooperation and patient triaging are necessary. Many health systems, following the management recommendations, used a patient-selection system, usually dividing breast-cancer patients into three or four surgical-time groups, from high (<2 weeks) to low surgical priority (>4 or up to >8 weeks), according to surgery urgency. Patients facing surgical complications (hematomas, abscesses, and flap ischemia) are of high surgical priority regardless of the COVID-19 urgency setting [10,11,19]. Patients completing neoadjuvant chemotherapy, breast cancer during pregnancy, T2 or N1 HR+/HER2- tumors, and triple-negative or HER2+ patients range from high to intermediate surgical priority, taking into consideration the COVID-19 urgency setting and alternative treatment options [8,9,10,11,12,14,15,18,19]. The excision of malignant recurrence, clinically low-risk primary disease, discordant biopsies likely to be malignant, and patients unable to receive neoadjuvant treatment are considered of intermediate priority [8,9,10,11,12,14,15,18,19]. All high-risk benign lesions, DCIS cases, discordant biopsies likely to be benign, re-excision surgeries, prophylactic operations, delayed sentinel lymph node biopsies, and primary-systemic-treatment-eligible patients are classified as low priority [10,11,19].

The type of surgical approach was also affected, with an increase in minimal, breast-conserving operations in order to avoid extensive surgeries and thereupon the risk of major complications, patient revisits, unnecessary hospitalizations, and prolonged hospital stay [6,8,9,14,16]. When mastectomy was performed, immediate breast reconstruction (IBR) was not the reconstructive method of choice; instead, delayed reconstruction was preferred in order to reduce surgical time and the risk of complications in many cases [12,16]. The axillary surgical approach does not seem to have been affected, although some technical considerations arose regarding the COVID-19 vaccination site and timing or the feasibility of dual tracer sentinel lymph node (SLN) mapping, creating space for possible alternatives [16,20,21].

Regarding radiotherapy and systemic therapy, following the same principles of in-person appointment and hospitalization reductions and resource retaining, several alterations were applied. Current practices and literature support that adjuvant radiotherapy initiation can be delayed for up to 3–6 months for selected patients, and hypofractionated radiotherapy schedules are preferred during periods of health-system overload since there seems to be no difference in terms of the therapeutic effect [7,9,10,13,15,22]. In general, radiation treatment was preserved for high-risk breast-cancer patients postoperatively and as palliative treatment for local or metastatic disease presenting with urgent or otherwise uncontrollable symptoms (bleeding mass, spinal cord compression, and symptomatic brain lesions), in addition to patients already on treatment [10,11].

Both adjuvant and neoadjuvant hormonal therapy (NET) for every stage HR positive tumors became widely suggested, as well as HER2-directed therapy for HER2+ disease, facilitating the delay of surgical treatment [2,6,8,9,13,14,16]. More specifically, primary hormonal therapy was considered acceptable in most of HR+/HER2- cases, especially post-menopausal women, in some countries, including those with N1 axillary disease, accompanied by neoadjuvant chemotherapy in high-risk patients, the selection of whom was facilitated by the use of genomic testing [5,6,9,12,13,14]. This practice can delay surgical treatment up to 6–12 months [11]. Wilke et al. report an additional 31% of the patients in their database receiving NET due to COVID-19, in contradiction to 6.9% receiving NET as the usual approach, highlighting the impact of the pandemic on this particular type of treatment [6]. Chemotherapeutic protocols were modified in order to avoid toxicity and chemotherapy (CMT)-related adverse effects (most importantly immunosuppression), with complete avoidance of CMT in selected patients, the use of longer interval regimens, universal-growth-factor support, and limited anthracycline and steroid use [9,10,11,13,15]. Genomic testing, even on biopsy specimens, was strongly encouraged for patient selection [6,9,13].

### 2.3. Challenges in Follow-Up

Managing patient visits after treatment initiation was also a field affected by the pandemic, as this part of caregiving is not considered as urgent and therefore was subject to limitations. In the absence of symptoms, routine follow-up and breast-imaging appointments were deferred, with a reduction in in-person visits up to 49.4% in some countries [10,12,13,23]. Telemedicine came up again as a solution to some cases, filling part of this void and cutting down the necessity for face-to-face appointments, while providing adequate healthcare services [10,23]. It was included as the recommended method for established cases without new issues, psychological support visits, and newly diagnosed non-invasive breast-cancer patients [11].

### 2.4. Challenges for the Patients

While healthcare personnel have often been found crushed under the pandemic’s suffocating burden, patients were not at all spared the psychological pressure imposed by this new threat either. The pandemic brought additional stressors to an already psychologically vulnerable population group, such as the fear of infection, especially for immunosuppressed patients; the fear of disease undertreatment and recurrence due to delays and changes in cancer therapies; and the logistics of scheduling and attending a screening, treatment, or follow-up appointment [1,17,24]. In addition, through restrictive measures and isolation, it deprived them of several supporting mechanisms: in-person communication and interaction, participation in group activities, and even receiving specialized help and care. Breast-cancer patients’ life became gloomier, as a Canadian study shows, with 63.9% of the participants declaring having experienced at least one COVID-19-related stressor and almost 40% showing clinical levels of concerns such as anxiety, insomnia, and depressive symptoms [24]. Promoting mental health and emotional stability for these patients is a principal priority per se but also serves as a way to ensure better compliance and engagement, avoiding screening or treatment drop outs that jeopardize patients’ outcomes [1,24]. Communication is key to overcoming these psychological obstacles, alleviating the frustration deriving from management alterations and providing needed information, counseling, and support, creating a safe environment for the patient, even when it is accomplished remotely or with the use of informational material [1,17].

### 2.5. Challenges in Breast-Surgery Education

The pandemic-induced shift from surgical to non-surgical breast-cancer treatments left breast surgical training in crisis too. The inevitable changes in clinical practice affected surgical trainees worldwide regardless of their training program duration or setting. Breast-surgery fellowships in particular, due to the short-time programs (usually 1 year), faced major interruptions.

The alterations that most affected postgraduate surgical education include the dramatic decrease in elective operations, the missing cases due to diagnostic delays, the reduced clinic and outpatient unit hours, and the trainees’ redeployment to COVID-19 related units [25,26,27,28]. The main result of these changes was a significant gap in hands-on, operative, and in-patient exposure. As stated by Kilgore et al., 43% of breast-surgery trainees incurred partial or complete deprivation of their time in the operating room [26]. In a prospective study conducted by a COVID-STAR collaborative study group among trainees of several surgical subspecialties, including breast surgery, the respondents reported a complete or >50% loss of their training regarding elective operating, emergency operating, and outpatient activity in 69.5%, 48%, and 67.3% of respondents, respectively, which affected their progression and perception of competence in their fields [28]. Moreover, reduced contact and communication with physicians of the same or relevant specialties in the context of COVID-19 risk-reduction measures hindered mentorship and peer guidance, further limiting gained experience.

Apart from the clinical aspects of surgical education, delays in clinical research; cancellations and disabled attendance in conferences; and postponements of certification or qualifying exams posed a threat to academic development [25,26,29].

As a result of educational and professional uncertainty, along with a fear of COVID-19 infection and transmission, stress levels were heightened; 81% of surgical trainees declared that their mental health was affected by the situation, and 93% of breast-surgery fellows admitted having faced increased stress [26,28]. The educational challenges hide a long-term breast-cancer management challenge since the deficient training at the present could lead to insufficient surgeons in the future, imperiling patients’ safety and outcomes [27].

## 3. Discussion

The ongoing COVID-19 pandemic has been a burdensome situation for healthcare systems, clinicians, and patients worldwide. Breast-cancer management is facing several obstacles inflicted by this pandemic in all of its aspects. Screening, patient diagnosis, treatment and follow-up are all parts of this multifaceted challenge and the most important questions arising concern how these difficulties are to be overcome and what will the impact of our decisions be—its extent and its contribution—on breast-cancer patients’ course.

In such unprecedented conditions, providing high-quality cancer care with compromised material, human, and even psychological reserves requires cautious and thorough decision making, which can be achieved only when based on well founded, evidence-based knowledge and uninterrupted multidisciplinary coordination. Consultations and guidelines elaborated by scientific groups aid this process and provide a base for consonant clinical practice, without limiting the opportunity for personalized care. Furthermore, establishing and supporting communication between caregivers and patients with detailed and comprehensible information about their disease, options, and COVID-19 risks seems to be an important step towards a safe and effective therapeutic plan.

It is apparent that the impact of the current situation and its subsequent alterations in breast-cancer management is still unknown, and further investigation is essential. Screening and diagnostic delays due to suspended screening programs and reduced referrals, as well as patient hesitancy, could contribute to a shift towards higher stage disease at the time of diagnosis, losses in diagnoses, and ultimately an increase in breast cancer mortality, a hypothesis that needs to be assessed. Treatment modifications, such as the rapid increase in neoadjuvant hormonal therapy use, surgery and radiotherapy delays, and systemic therapy regimen alterations, are another field of evaluation, in order to define the safety and efficacy of COVID-19-driven management decisions and acquire valuable data on the impact of the pandemic on breast-cancer patients’ outcomes. Finally, although the changes implemented are considered as resource-sparing, the aftermath of breast-cancer screening and treatment distortions may still not favor health systems’ sustainability. It is of value to take into consideration whether missing early-stage disease, delaying surgery and performing two-step surgeries instead of one (as in the case of avoided immediate breast reconstruction operations) could ultimately increase the cost of breast-cancer care.

Another topic yet to be clarified is the actual relationship between COVID-19 and breast cancer in order to define the level of patients’ risk and include it in decision making. The existing literature suggests that breast cancer per se is not a major contributor to COVID-19 mortality, nor is its treatment, and COVID-19 infection outcomes in these patients are mostly affected by patient’s comorbidities [7,14,30]. Thus, it would be reasonable to redefine the existing modifications or the population that they concern, considering their application only for high-risk patients.

Despite being a first-line therapy for many cases, breast-cancer surgery is one of the fields receiving the most extensive changes and reductions for the sake of hospital operations and resource preservation. It should be noted though that the majority of oncological breast surgeries require short operation times and a limited hospital stay and have almost no need for ICU beds [12]. The rates of complication and hospital readmissions are low, especially when an IBR is not performed, and postoperative follow-up is usually not demanding [12]. Therefore, the need for strict surgery avoidance may be questioned, and the possibility of breast-cancer surgery regaining its place is a topic to be discussed.

Within difficulty lies opportunity, and this dire situation seems to be no exception to that. The urgent and massive need for clinical practice rescheduling received an immediate response from the scientific community, adjusting to the new setting effectively and responsibly in order to maintain the standard of care. The readiness and responsiveness are a promising sign, as are some of the decisions that the pandemic forced us to dare to make, including instituting the new therapeutic approaches and alternative ways of caregiving, such as the establishment of telemedicine, a tool that is going to be useful in the post-COVID era too. A careful interpretation of the new data acquired during this period could hopefully fructify in terms of progress.

## 4. Conclusions

The COVID-19 pandemic and the induced healthcare crisis enforced an era of shortages in caregiving, due to the massive needs in human and material resources and hospital beds, as well as in patients’ wellbeing and mental health as a result of the restrictive measures imposed. In this hostile setting, breast-cancer management faced challenges at multiple levels; from screening and prevention, to diagnosis, treatment, and follow-up, struggling at each step to deliver the highest possible quality of care. Responding to these conditions included delays and postponements, mostly for the diagnostic part and considerable or minor alterations for the therapeutic plans, with two major points regarding this field: the rise and extensive use of neoadjuvant hormonal therapy and the significant reduction in the first-line surgical approach. These changes in therapeutic strategy remain to be evaluated.
